# [Corrigendum] Tumor necrosis factor-α triggers opposing signals in head and neck squamous cell carcinoma and induces apoptosis via mitochondrial- and non-mitochondrial-dependent pathways

**DOI:** 10.3892/ijo.2026.5888

**Published:** 2026-04-27

**Authors:** Denis Selimovic, Renate U. Wahl, Emmanuelle Ruiz, Rizwan Aslam, Thomas W. Flanagan, Sofie-Yasmin Hassan, Simeon Santourlidis, Youssef Haikel, Paul Friedlander, Mosaad Megahed, Emad Kandil, Mohamed Hassan

Int J Oncol 55: 1324-1338, 2019; DOI: 10.3892/ijo.2019.4900

Following the publication of the above article, an interested reader drew to the Editor's attention that a number of western blots in the paper appeared to contain incorrectly assembled data. First, comparing the F. Length (full-length) Caspase 3 blots in Fig. 1F with the p-ASK1 blots in Fig. 2C revealed that they were remarkably similar after horizontally flipping one set of the bands. In addition, the cytochrome *c* (Cyt. C) bands in Fig. 1F were similarly found to be remarkably similar to the IκBα blots in Fig. 2A, again after horizontal flipping of one set of the bands. Finally, it was noted that the β-actin bands in Fig. 5C were very similar to the blots included in Fig. 3B to show the Tom20 data.

After having examined the raw data underling these figures (which were also presented to the Editorial Office for our inspection), the authors realized that data in Figs. 1, 2, 3 and 5 were inadvertently assembled incorrectly in these figures. The revised versions of Figs. 1, 2, 3 and 5 are shown on the subsequent six pages. Specifically, in Fig. 1F, the blot for Cyt. C was incorrectly chosen; in Fig. 2A, the blot for IkBα was incorrectly chosen; in Fig. 3B, the blot for Tom20 was incorrectly chosen; and in Fig. 5C, the β-actin blots shown to represent the gels for both the treated and control CLS-354 and RPMI 2650 cells were incorrectly placed in this figure. These data have all been replaced with the correct data in the figures shown subsequently in this Corrigendum.

The authors regret that the errors in Figs. 1F, 2A, 2C, 3B and 5C went unnoticed in the published versions of these figures in this paper, although note that these errors did not influence either the validity of the published data or the conclusions described in the article. The authors are grateful to the Editor of *International Journal of Oncology* for allowing them the opportunity to publish this Corrigendum. All the authors agree with the publication of this Corrigendum, and apologize to the readership for any inconvenience caused.

## Figures and Tables

**Figure 1:**
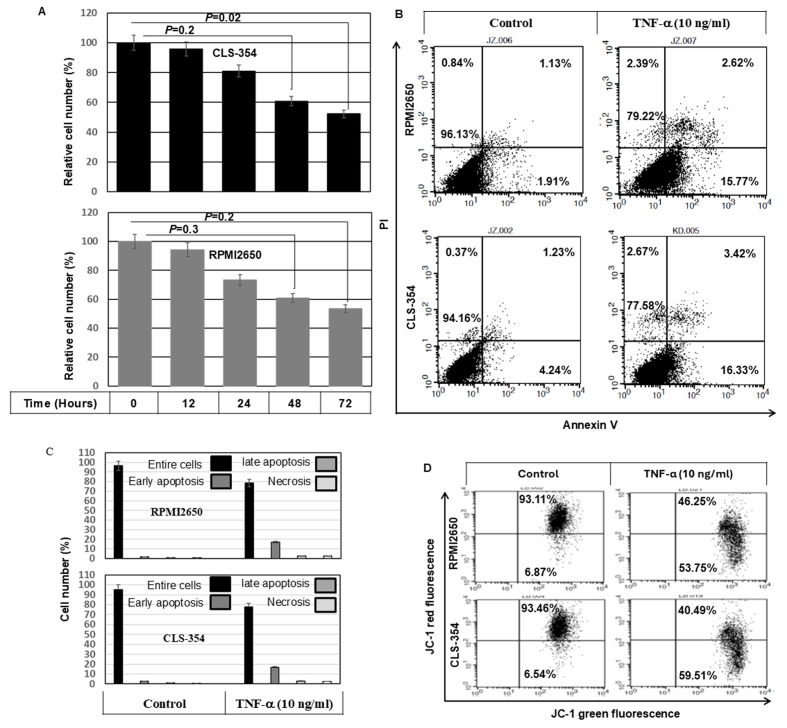

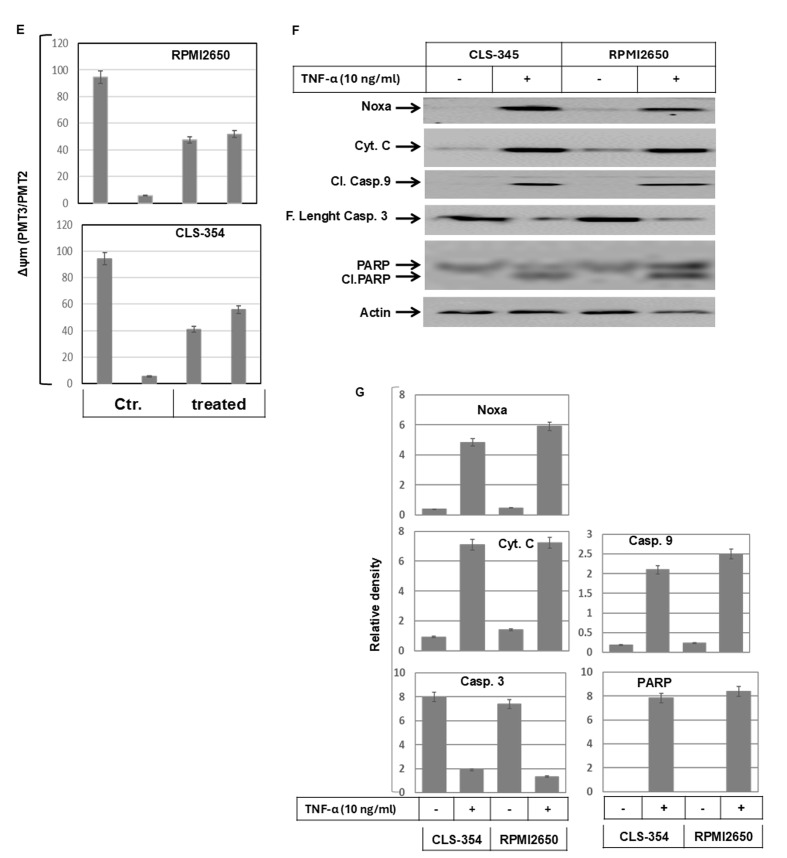
(A) Time course-dependent inhibition of the growth rate of head and neck squamous cell carcinoma (HNSCC) cells in response to exposure to TNF-α. Relative cell number (%) assessed by MTT assay following the exposure of the HNSCC cell lines, CLS-345 and RPMI2650, to TNF-α (10 ng/ml) for regulated time intervals up to 72 h. Data are presented as the means ± SD (n=4). ^*^P<0.05, significantly different from the control as shown by ANOVA and Dunnett's test. (B) Flow cytometric analysis using Annexin V/propidium iodide (PI) staining demonstrating the TNF-α-induced apoptosis of the HNSCC cell lines, CLS-345 and RPMI2650. (C) Data of of Annexin V/PI are presented as the means ± SD (n=3). ^*^P<0.05, significantly different from the control as shown by ANOVA and Dunnett's test. (D) Flow cytometric analysis using JC-1 staining demonstrating the loss of mitochondrial membrane potential (Δψm) in TNF-α-treated cells. (E) Data of JC-1 staining are presented as the means ± SD (n=3). In each treatment group, the first one of the two bars represents the portion of the cells that do not show loss of mitochondrial membrane potential, while the second bar represents the portion of cells that show the loss of mitochondrial membrane potential. ^*^P<0.05, significantly different from the control as shown by ANOVA and Dunnett's test. (F) Western blot analysis demonstrating the induction of Noxa expression, the release of cytochrome *c* (Cyt c), and the cleavage of caspase-9, caspase-3 and PARP in response to the treatment of the HNSCC cell lines, CLS-345 and RPMI2650, with TNF-α for 48 h. Actin was used as an internal control for loading and transfer. (G) Analyses of band intensity on films are presented as the relative ratio of Noxa to actin, released Cyt c to actin, cleaved caspase-9 (Cl.Casp.9) to actin, cleaved caspase-3 (Cl.casp.3) to actin and cleaved PARP (Cl.PARP) to actin. Bars represent the means ± SD (n=3). ^*^P<0.05 vs. control.

**Figure 2:**
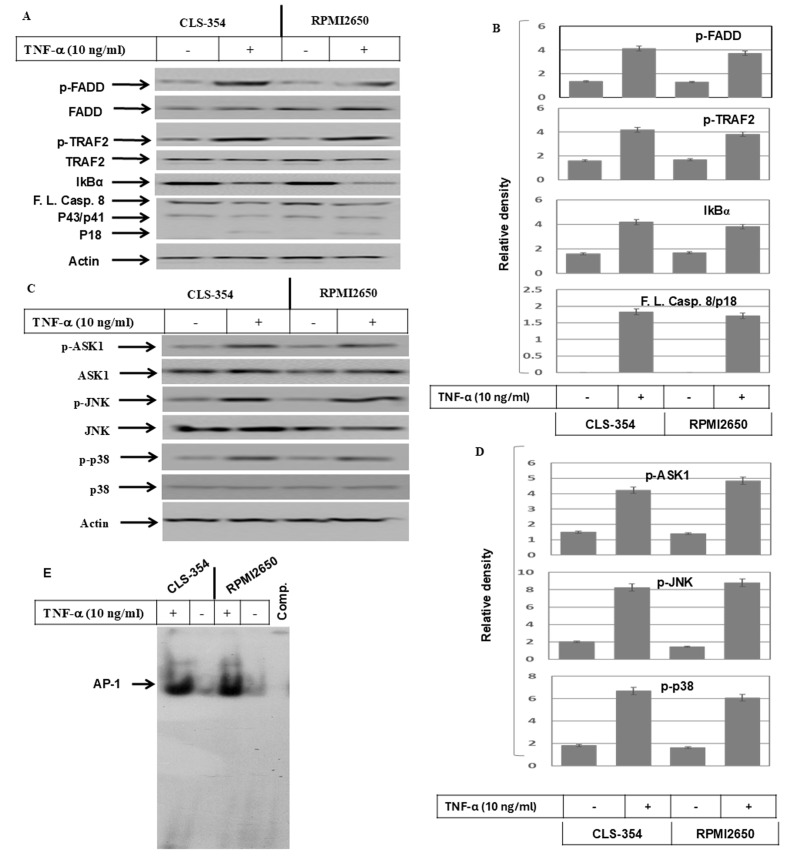

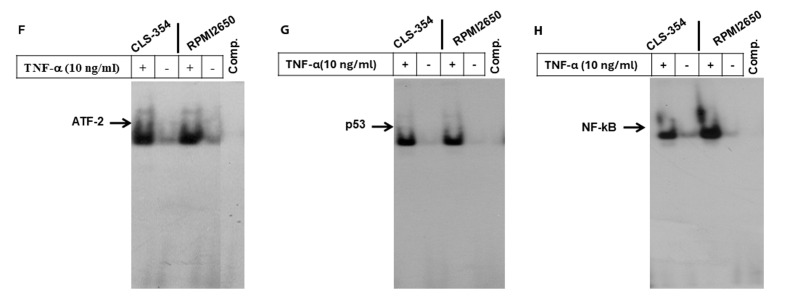
(A) Western blot analysis demonstrating the phosphorylation of FADD and TRAF2 proteins, and the degradation of IκBα and the cleavage of caspase-8 in response to the exposure of head and neck squamous cell carcinoma (HNSCC) cell lines, CLS-345 and RPMI2650, to TNF-α. Actin was used as an internal control for loading and transfer. (B) Analyses of band intensity on films are presented as the relative ratio of p-FADD to actin, p-TRAF2 to actin, IκBα to actin and cleaved caspase-8/p18 (Cl.casp.8/p18) to actin. Bars represent the means ± SD (n=3). ^*^P<0.05 vs. control. (C) Western blot analysis demonstrating the phosphorylation of ASK1, JNK and p38 kinase in response to the exposure of the HNSCC cell lines, CLS-345 and RPMI2650, to TNF-α. Actin was used as an internal control for loading and transfer. (D) Analyses of band intensity on films are presented as the relative ratio of p-ASK1 to actin, p-JNK to actin and p-p38 to actin. Bars represent the means ± SD (n=3). *P<0.05 vs. control. EMSA demonstrates the enhancement of the DNA-binding activity of the transcription factor, (E) AP-1. EMSA demonstrates the enhancement of the DNA-binding activity of the transcription factors, (F) ATF-2, (G) p53 and (H) NF-κB in response to the treatment of the HNSCC cell lines, CLS-345 and RPMI2650, with TNF-α. Data are representative of 3 independent experiments.

**Figure 3:**
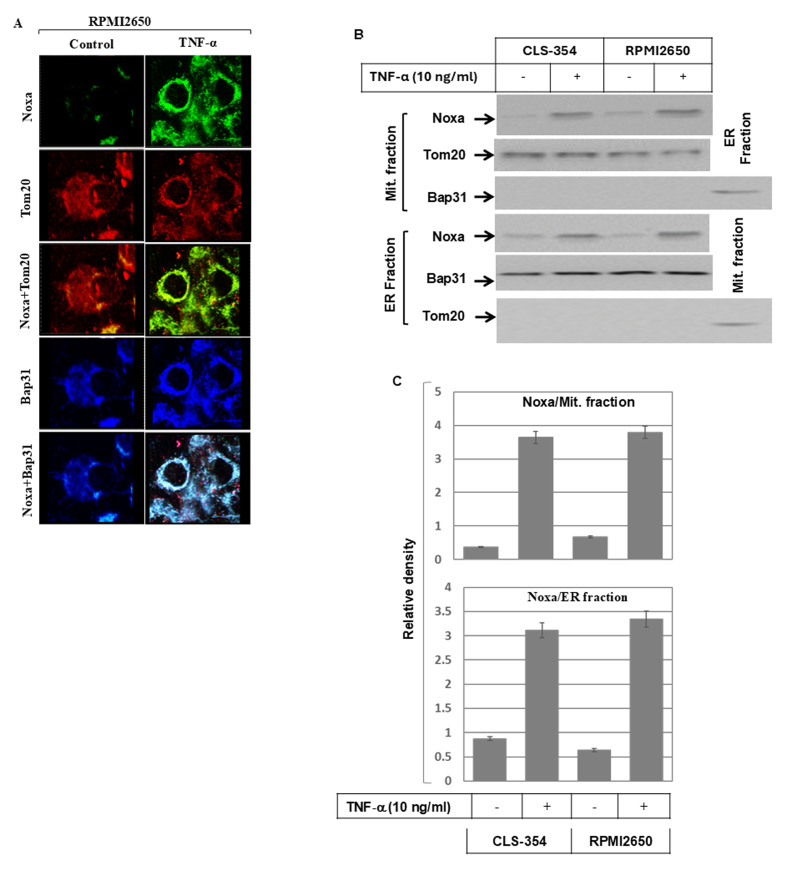
Subcellular localization of Noxa protein to both the mitochondria and endoplasmic reticulum (ER) in TNF-α-treated and control RPMI2650 cells. (A) Immunofluorescence (IF) staining: RPMI2650 cells were treated with TNF-α for 48 h prior to staining with anti-Noxa, Tom20 (mitochondrial marker) and Bap31 (ER marker). The subcellular localization of Noxa (green) to mitochondria (red) and the overlay of Noxa with Tom20 staining demonstrates the localization of Noxa to the mitochondria (yellow), when compared with control cells. The localization of Noxa (green) to ER (blue) and the overlay of Noxa with Bap31 staining demonstrates the localization of Noxa to ER (turquoise), when compared with control cells. (B) Western blot analysis using the mitochondrial fraction (Mit.fraction) and ER fraction from both the CLS-354 and RPMI2650 cells following treatment with TNF-α for 48 h. The detection of Noxa in the mitochondrial and ER fractions of the CLS-354 and RPMI2650 cells following treatment with TNF-α was used to confirm the localization of Noxa protein to both the mitochondria and ER. The purity of both the mitochondrial and ER fractions was verified by the detection of the mitochondrial protein, Tom20, in the mitochondrial fraction and the detection of Bap31 in the ER fraction. (C) Analyses of band intensity on films are presented as the relative ratio of Noxa to Tom20 in the mitochondrial fraction and Noxa to Bap31 in the ER fraction. Bars represent the means ± SD (n=3); ^*^P<0.05 vs. control.

**Figure 5:**
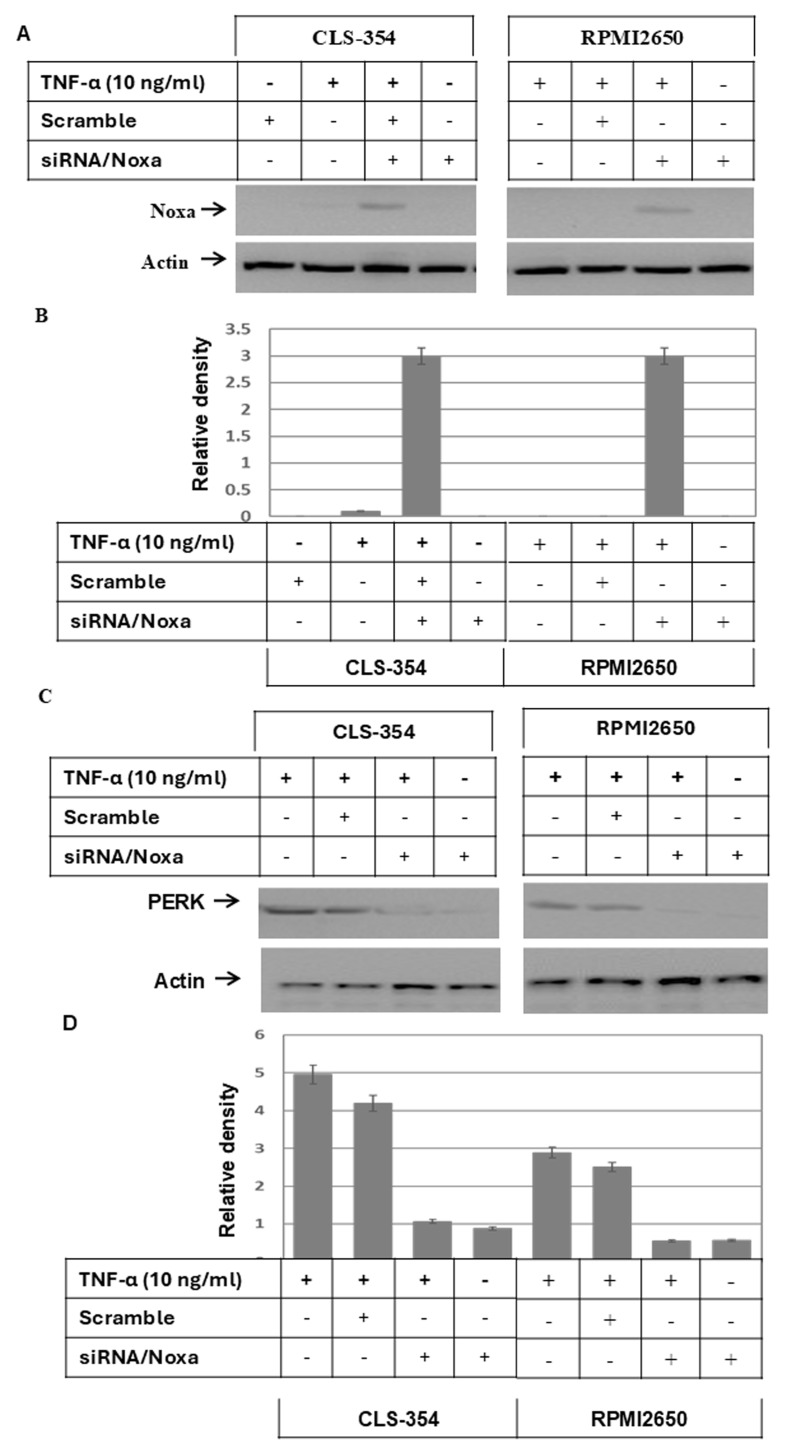

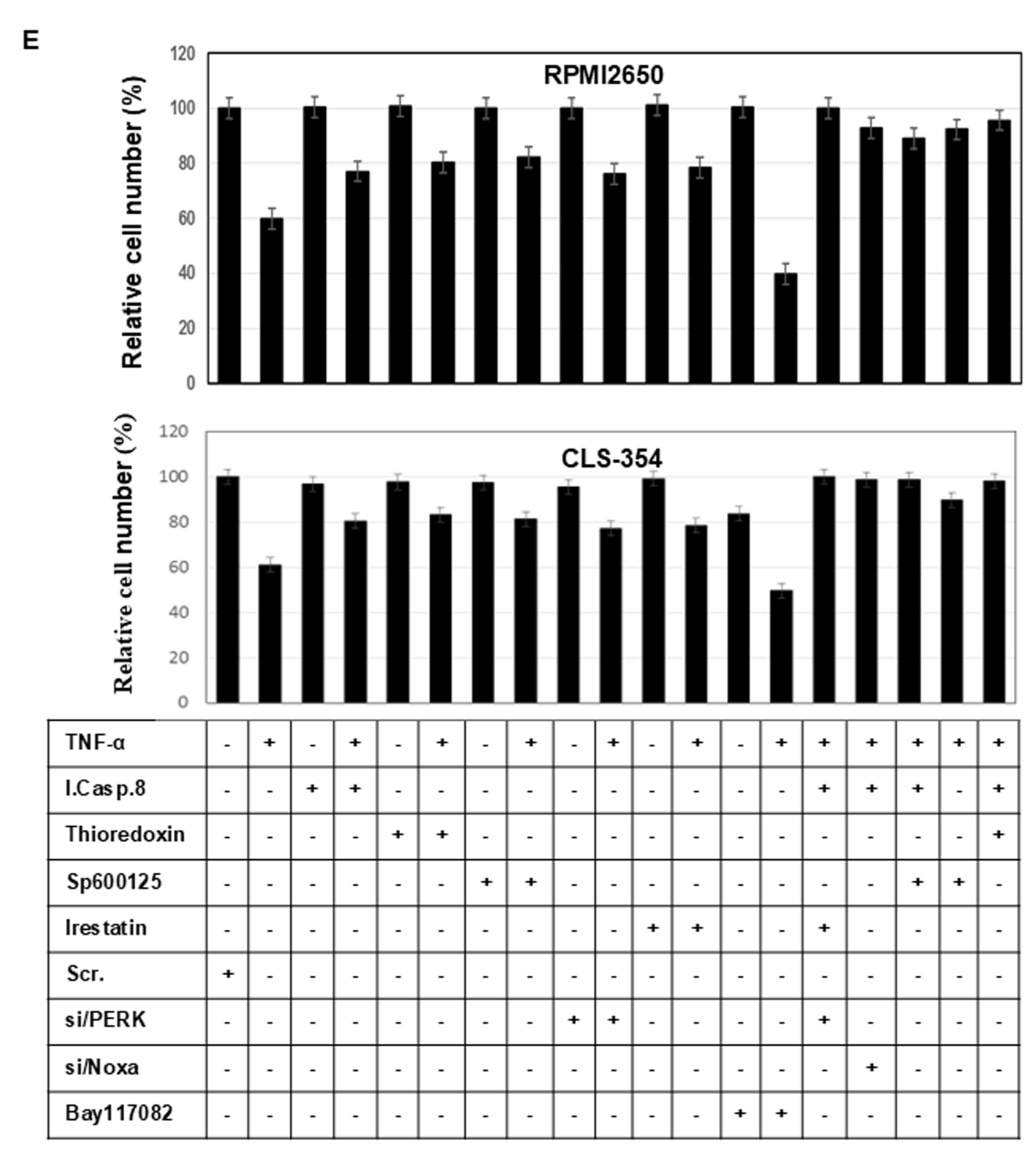
(A) Western blot analysis demonstrating the knockdown of Noxa protein by its specific siRNA (si/Noxa) in CLS-354 and RPMI2650 cells treated with TNF-α (10 ng/ml). (B) Analyses of band intensity on films are presented as the relative ratio of reduced Noxa to actin. Bars represent the means ± SD (n=3). ^*^P<0.05 vs. control. (C) Western blot analysis demonstrating the knockdown of PERK protein by its specific siRNA (si/PERK). Actin was used as an internal control for loading and transfer. (D) Analyses of band intensity on films are presented as the relative ratio of reduced PERK expression to actin. Bars represent the means ± SD (n=3). ^*^P<0.05 vs. control. (E) MTT assay demonstrating the inhibition of TNF-α-induced cell death of RPMI2650 (upper panel) and CLS-345 (lower panel) via inhibitors of caspase-8 (I.Casp.8, 20 *μ*M), ASK1 (thioredoxin, 1 nM), JNK (SP600125, 10 *μ*M), IRE1α (Irestatin, 25 *μ*M), or by the knockdown of PERK by its specific siRNA (si/PERK). The complete abrogation of the TNF-α-induced cell death of head and neck squamous cell carcinoma (HNSCC) cell lines was noted by the knockdown of Noxa by its specific siRNA (si/Noxa) or the combination of ASK1 and caspase-8 inhibitors. Pretreatment of the cells with NF-κB inhibitor (Bay11-7982, 5 *μ*M) promoted TNF-α-induced cell death as evidenced by MTT assay. Data are presented as the means ± SD (n=3) performed in quadruplicate.

